# Polynaphthylimide–Azomethines Containing Triphenylamine or Carbazole Moieties with Tuned Optoelectronic Properties through Molecular Design

**DOI:** 10.3390/molecules27185761

**Published:** 2022-09-06

**Authors:** Marius Soroceanu, Catalin-Paul Constantin, Mariana-Dana Damaceanu

**Affiliations:** “Petru Poni” Institute of Macromolecular Chemistry, Aleea Gr. Ghica Voda 41A, 700487 Iasi, Romania

**Keywords:** polyazomethines, naphthylimide, carbazole, triphenylamine, opto-electronic behavior, bandgap energy

## Abstract

Polyazomethines containing electron-donor triphenylamine (TPA) or carbazole (Cbz) and electron-acceptor naphthyl(di)imide were synthesized and investigated with regard to thermal, optical and electronic features, with a focus on their modulation by molecular design. The polycondesation of an imido-based diamine with a Cbz- or TPA-based dialdehyde led to donor-acceptor polymers with good thermostability, up to 318 °C. These displayed good solubility in organic solvents, which enabled easy polymer processability in thin films with different molecular assemblies. The molecular order improved the charge carrier’s mobility, with a direct impact on the bandgap energy. The optical properties studied by UV–Vis absorption and fluorescence experiments showed solvent-dependence, characteristic for donor-acceptor systems. The structural parameters exerted a strong influence on the light-emissive behavior, with the prevalence of intrinsic or intramolecular charge transfer fluorescence contingent on the donor-acceptor strength and polymer geometry. All polymers showed good electroactivity, supporting both electrons and holes transport. The exchange of Cbz with TPA proved to be an efficient tool with which to decrease the bandgap energy, while that of naphthyl(di)imide with bis(naphthylimide) was beneficial for fluorescence enhancement. This study may contribute to a deeper understanding of the physico-chemistry of electronic materials so as to make them more competitive in the newest energy-related or other optoelectronic devices.

## 1. Introduction

Organic electronics is one of the evolving technologies enabling development of eco-friendly, lightweight, flexible and low-cost devices with versatile functionalities not attained by silicon or other inorganic materials—that is, electronic devices that bend, twist, and conform to any surface [1]. In this field, π-conjugated polymers attracted much interest, being the subject of research for many years, due to their widespread use as active components in various devices, including photovoltaic cells, thin-film transistors or electroluminescent diodes [2,3]. However, these devices are still in infancy relative to the commercial ones and need to overcome key research challenges towards a more innovative and sustainable approach to developing our electronic world.

The main advantage encountered in conducting polymers relates to the possibility of fine tuning the molecular structure through the organic synthetic strategies so as to achieve desired and adjustable optoelectronic properties [4]. Apart from this, the simple synthetic procedure and the reduced cost made them attractive replacements for the outstanding semiconductors despite their still-low performance in many applications. They are also candidates for wet processing techniques, forming homogeneous thin films of good quality suitable for flexible electronic devices [5].

In recent years, azomethine (-CH=N-)-based conjugated polymers emerged as promising materials in different optoelectronic applications [6], being considered appropriate alternatives to π-conjugated polymers containing vinylene (-C=C-) bonds [7]. Conjugated aromatic polyazomethines have demonstrated attractive electronic, optoelectronic and thermal properties that can be readily modified depending on the molecular design. These have found a plethora of applications, such as electroluminescent materials in light-emitting devices, photonic memories or electrochromic materials, hole transporting materials in solar cells or active layers in organic field-effect transistors [8,9,10,11,12,13,14]. However, a challenge that still faces the development of polyazomethines is to obtain solution-processable, highly conducting or fluorescent materials in a neutral state [15,16,17]. In this regard, there is still a need to incorporate new functional groups in the main chains to attain both improved solubility and innovative physicochemical properties, so as to make them compatible with current optoelectronic devices. Some examples are reported in [18,19], where Schiff base-derived materials with innovative aggregate-induced emission, mechanochromic or photochromic characteristics were developed, which can provide a guideline towards sustainable polyazomethines for use in advanced technologies.

One approach in this regard envisages the incorporation of triphenylamine (TPA) moieties in polyazomethines. This propeller shape heteroaromatic unit endows the polymers with both good processability and electroactivity, besides other useful characteristics, owing to its redox activity, fluorescence or ferromagnetic behavior. It was widely used as hole transporting core, as well as a building block in small molecules, dendrimers, and polymers [8,20,21,22]. Another attractive functional group is carbazole, particularly because various substituents can be attached to its nitrogen atom, while the aromatic core can be easily substituted, allowing the tuning of its optoelectronic properties. Polymers containing carbazole are highly thermostable and display good electro- and photoactive properties induced by their hole-transporting ability and high absorption in the UV domain. Due to these characteristics, such polymers were used in smart windows, solar cells, electroluminescent devices, biosensors, and other related applications [23].

Embedding a hole-transporting unit along with an electron accepting one separated by a π-spacer in one polymer chain can generate donor-acceptor systems that can efficiently meet the demands of optoelectronic devices [24]. The properties of these materials can be readily adjusted by exchanging the donor or the acceptor according to the need, so that a fine tuning of the HOMO, LUMO and bandgap energies, as well as other optoelectronic properties, can be reached. Tetracarboxylic diimides, especially those with six-membered imide rings like naphthalene(di)imides, are appropriate candidates for donor-acceptor systems, being known as electron-deficient moieties which can support the electrons’ transport while they are very stable in air [25]. Besides, these units endow the materials with important properties, such as high thermal stability, optical response, chemical and oxidation resistance and good capability to pack into organized supramolecular structures [26,27].

Among the large family of donor-acceptor systems, polymers containing the strong electron acceptor naphthyl(di)imide core directly connected to the azomethine unit that can enable an easy electron flow to the donor unit are still unknown. They can subscribe to the family of materials with the ability to fine tune the physicochemical and (opto)electronic characteristics, facilitated by synthetic chemistry involving azomethine bond generation. Recently, we have reported our first attempt to prepare oligomer systems with such molecular design [28], and since we found their characteristics attractive for optoelectronic applications, here we extended this structural pattern to polymers. The scope was to survey the possibility of fine tuning the HOMO, LUMO and bandgap energies, as well as the florescence properties through an extended π-system and through structural parameters. Our target was also to overcome one of the challenges encountered with these aromatic polymers, which is low solubility due to strong intermolecular interactions, so as to be conveniently processed into thin layers from solutions by using easily accessible solvents.

Thus, this study is focused on the development of donor-acceptor polymers in which the azomethine is the π-bridge between the electron acceptor naphthyl(di)imide and electron donor TPA or Cbz. The correlation between structure and targeted properties was investigated in terms of film processing capability and morphology, thermal stability, photo-optical, electrochemical and electronic properties. The effect of electron-donor or electron-acceptor exchange on the overall physicochemical characteristics of these polymers was particularly investigated.

## 2. Experimental

### 2.1. Starting Materials

The structure of the diamine and dialdehyde monomers (**M1**–**M4**), which were allowed to react by polycondensation reaction to produce polyazomethines **P1**–**P4**, are illustrated in Figure S1 (Supporting Information, SI). Naphthalene-N,N’-bis(imido-amine) (**M1**) was prepared following the procedure reported in [19]. 1,1-Dichloro-2,2-bis(1,8-dicarboxynaphthalene-N-imido-amine)ethylene (**M2**) was obtained according to reference [21]. 9-(2-Ethylhexyl)carbazole-3,6-dicarboxaldehyde (**M3**) and 4,4′-diformyltriphenylamine (**M4**) were purchased from Sigma-Aldrich and used as received, with no further purification. Other reagents and materials were provided by various commercial sources and used without purification.

### 2.2. Polymers Synthesis

**Scheme 1:**
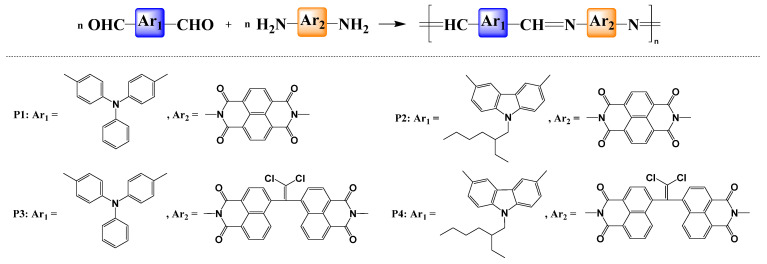
Synthetic way to polyazomethines **P1**–**P4**.

Four donor-acceptor polymers containing azomethine linkages were prepared by the well-known polycondensation procedure [8,10], starting from aromatic diamines containing naphthyl(di)imide unit (**M1**, **M2**) and 9-(2-ethylhexyl)carbazole-3,6-dicarboxaldehyde (**M3**) or 4,4′-diformyltriphenylamine (**M4**), as shown in Scheme 1.

The reactions were carried out either in CHCl_3_ (**P1** and **P2**, based on **M1** diamine) or DMF (**P3** and **P4**, based on **M2** diamine) at a concentration of 10% total solids, under a nitrogen stream and at 60 °C (in CHCl_3_) or 120 °C (in DMF) for 24 h. In the case of **P1** and **P2**, the polycondensation reaction was performed under catalytic conditions in the presence of trifluoroacetic acid (TFA). The following example illustrates the general procedure. 

In a 25 mL vacuum-dried Schlenk flask purged with nitrogen and equipped with a magnetic stirrer, the diamine **M1** (88.23 mg, 0.298 mmol) and CHCl_3_ (2 mL) as solvent were placed. Then, dicarbaldehyde **M3** (100 mg, 0.298 mmol) was added to the resulting suspension, being followed by the dropping of 20 µL TFA. Then, the reaction mixture was heated under stirring and nitrogen at 60 °C for 24 h. When the temperature reached 60 °C, the solution color turned from dark green to dark red. Upon cooling to room temperature, the obtained reaction mixture was poured into dry methanol to precipitate **P2** as a dark-red solid. The purification was performed by multiple washing of the polymer with hot methanol. After drying in an oven at 100 °C for 6 h, **P2** was obtained as a light- red powder, with a reaction yield of 85.5%. The other polymers were obtained as brown (**P1**), yellow (**P3**) or dark-yellow (**P4**) powder, with a reaction yield of 69.1%, 87% or 90.2%, respectively.

**P1**, 1H NMR (DMSO-d6, 400.13 MHz, δ ppm): 8.74–8.72 (m, 6H), 7.96–7.83 (dd, 4H), 7.50–7.47 (t, 2H), 7.33–7.24 (m, 5H), 7.18–7.16 (d, 2H).
**P2**, 1H NMR (DMSO-d6, 400.13 MHz, δ ppm): 8.89–8.68 (m, 6H), 8.22–8.20 (dd, 4H), 8.06 (s, 2H), 7.86–7.83 (m, 2H), 4.42–4.39 (m, 2H), 2.04 (s, 1H), 1.33–1.19 (m, 8H), 0.88–0.77 (m, 6H).
**P3**, 1H NMR (DMSO-d6, 400.13 MHz, δ ppm): 8.97–8.91 (m, 2H), 8.64–8.49 (m, 4H), 8.13–8.19 (m, 2H), 7.92–7.81 (m, 6H), 7.47–7.45 (d, 2H), 7.29–7.14 (m, 7H).
**P4**, 1H NMR (CDCl3, 400.13 MHz, δ ppm): 8.84–8.50 (m, 8H), 8.30–8.22 (m, 2H), 8.11–7.95 (m, 2H), 7.71–7.43 (m, 4H), 4.34–4.21 (m, 2H), 2.10 (s, 1H), 1.40–1.25 (m, 8H), 0.97–0.86 (m, 6H).

### 2.3. Preparation of Polymer Films (Coatings)

Thin polymer films were prepared at room temperature by using two techniques: spin-coating and drop-casting. Before deposition, all plates were firstly cleaned with deionized water and then with toluene and isopropanol in an ultrasound bath. Glass, quartz and indium tin oxide (ITO)-coated glass substrates were used as plates to coat thin polymer layers from chloroform (CHCl_3_) solutions with a concentration of 1%. In the case of the spin-coated films, the rotational speed was set to 1000 rpm, whereas the film deposition time was maintained for 30 s. The solvent evaporation was allowed to occur at room temperature under slight air ventilation. The as-obtained films were subjected to various investigations to assess the morphological, optical and electrochemical characteristics.

### 2.4. Measurements

The 1H-NMR spectra of the polymers were obtained with a Bruker Advance III 400 spectrometer (Bruker, Rheinstetten, Germany) operating at 400.13 MHz for 1H nuclei. The chemical shifts of the protons are provided in δ units (ppm) against the residual peak of the solvent. 

The Fourier-transform infrared spectroscopy (FTIR) experiments were carried out on a FT-IR Vertex 70 Spectrophotometer (Bruker, Ettlingen, Germany), by using KBr pellets. 

Thermogravimetric analysis (TGA) was recorded on the thermobalance STA 449F1 Jupiter (Netzsch, Selb, Germany) by heating the sample in an open Al_2_O_3_ crucible in nitrogen environment from 25 °C to 700 °C. The heating rate was set at 10 °C/min, and Al_2_O_3_ was used as reference material.

The UV–Vis absorption spectra were registered on an Analytik Jena-Specord 210 PLUS spectrophotometer (Analytik Jena, Jena, Germany) operating between 200 and 1100 nm by using dilute polymer solutions (approx. 10^−5^ M) or thin polymer films deposited on quartz substrates. 

Perkin Elmer LS 55 apparatus (PerkinElmer, Inc./UK Model LS 55, Waltham, MA, USA) was used for the acquisition of the fluorescence spectra of the polymers on similar samples used in UV–Vis absorption experiments. 

The morphology of the thin films spin-coated or drop-cast onto glass supports was investigated by scanning electron microscopy (SEM) using a Quanta 200 ESEM (FEI Company Hillsboro, OR, USA). 

The thicknesses of the films were estimated by KLA Tencor D500 contact profiler (D500, KLA Tencor, Milpitas, CA, USA).

The electrochemical characteristics of the polymers were assessed on the basis of cyclic voltammetry (CV) experiments, which were carried out on a Potentiostat-Galvanostat (PG581, Uniscan Instruments, Buxton, United Kingdom). The electrochemical cell was composed of three electrodes: a reference electrode (Ag/Ag^+^), an auxiliary electrode (platinum wire) and a working electrode (analyte-coated ITO glass). The cell was completed with an electrolyte salt (tetrabuthylammonium perchlorate—TBAP) dissolved in acetonitrile (ACN), at a concentration of 0.1 M, and then purged with a gentle flux of N_2_. The CV curves were recorded at room temperature, at a scan rate of 50 mV/s, while ferrocene was employed as the external reference for calibration (E_onset_ = 0.34 V in ACN vs. Ag/Ag^+^).

## 3. Results and Discussions

### 3.1. Synthesis and Structural Characterization

Carbazole (Cbz) or triphenylamine (TPA)—containing electron donors along with electron acceptors based on naphthyl(di)imide were used as the main building blocks to obtain polymers with azomethine as a π-conjugated bridge. The monomers were reacted by polycondensation at the 1:1 stoichiometry ratio in CHCl_3_, in the presence of trifluoroacetic acid as catalyst, or in DMF, without catalyst, as described in the Experimental sections. The different experimental conditions were adjusted so as to achieve satisfactory reaction yields as well as to keep the final polyazomethine in solution.

The formed polyazomethines were fully soluble in chlorinated solvents like chloroform (CHCl_3_) and methylene chloride (CH_2_Cl_2_) and partly soluble in tetrahydrofurane (THF), dimethylsolfoxide (DMSO) and dimethylformamide (DMF). However, **P3** and **P4** displayed solubility in these solvents at a higher concentration compared to **P1** and **P2,** owing to the higher chain flexibility induced by the CCl_2_ kinking center, leading to polyazomethines with more twisted chain conformations. Generally, this is a significantly improved solubility compared to that of fully aromatic polyazomethines, well explained by the non-coplanarity of the three aromatic rings in TPA units or long aliphatic side chains grafted on carbazole moieties, leading to higher distances between macromolecules and reduced physical bonding. The good solubility of the present polymers enabled their processing by wet methods, including spin-coating and spin-casting, so as to obtain thin films suitable for optoelectronic applications.

The investigated naphthylimide-based polyazomethines displayed low molar masses when investigated by GPC in CHCl_3_, suggesting their oligomeric nature. Thus, M_w_ was found in the range of 1540–5680 g/mol, whereas M_n_ ranged between 1280 and 3290 g/mol (Table 1).

They all displayed a polydispersity below 1.9, which is characteristic for polymers obtained by the polycondensation method. Although **P3** and **P4** exhibited higher molecular weights compared to **P1** and **P2**, this was expected due to the higher molar mass of the structural unit of **P3** and **P4** compared to **P1** and **P2**. Also, it was observed that Cbz-derived polymers **P2** and **P4** displayed slightly higher molecular weights compared to their counterparts based on TPA, **P1** and **P3**, which can be explained by the better reactivity of dialdehyde **M3** relative to that of **M4** towards diamines **M1** and **M2**. However, this is only a crude estimation of the molar masses of the present polymers, since standards of polystyrene were used for calibration, with significant structural differences compared to our polymers.

The successful accomplishment of the polymerization reaction was assessed by 1H- NMR and FTIR analyses. The ^1^H–NMR spectra of polyazomethines **P1**–**P4** are shown in Figure S2. All proton chemical shifts assignments were made by comparing the spectrum of the polymer with those of the starting monomers, as representatively illustrated in Figure S2b for **P2**. One of the main characteristics of the synthesized polyazomethines relates to the presence of the end-capped aldehyde groups, mostly due to low reactivity of the naphthyl(di)imide-based diamines. As highlighted in the spectra of **P2** and corresponding monomers, there is a small shift, up to 0.02 ppm (8.15 Hz), between the aldehyde proton signal from the polymers and that from the starting aldehyde counterpart. Moreover, the absence of the chemical shifts corresponding to NH_2_ protons at 5.86 ppm (**M1**) and 5.78 ppm (**M2**) proved the total incorporation of the diamine segments in the structure of the obtained polymers. Under these circumstances, the azomethine proton signal as representative proton signal for this class of polymers could be accurately identified at 8.72 ppm for **P1** and **P2**, and 8.62 ppm for **P3**. In the case of **P4**, this specific signal was found overlapped with other aromatic protons signals. The aromatic protons belonging to TPA and Cbz structures were found in the region 8–7 ppm (**P1** and **P3**) or 9–7 ppm (**P2** and **P4**). In the case of Cbz-based polymers (**P2** and **P4**), the protons belonging to the aliphatic side groups were clearly evidenced by the signals at approx. 4.29, 2.10, 1.40–1.27, 0.97 and 0.87/0.92 ppm.

**Figure 1:**
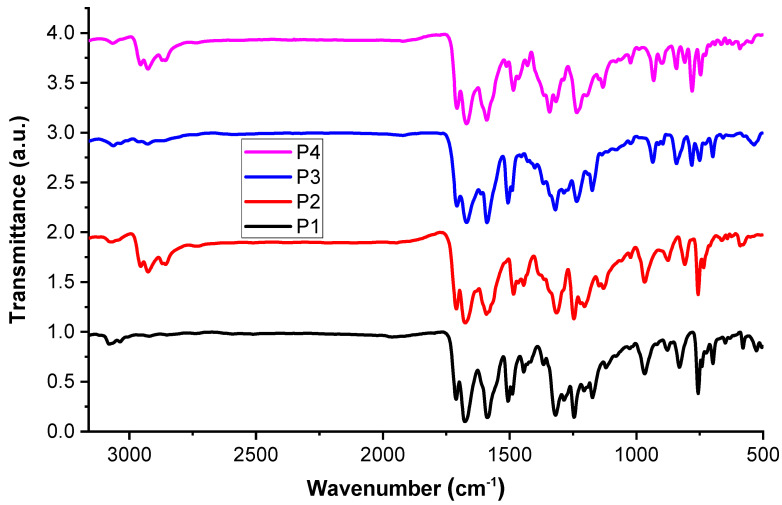
FT-IR spectra of polymers **P1**–**P4** containing naphthyl(di)imide units.

FTIR spectra brought further evidence on the appropriate structure of the synthesized polymers. In these spectra, the azomethine units gave strong bands overlapped with those generated by the symmetrical stretch of the C=O bond in the naphthyl(di)imide in the range of 1678–1670 cm^−1^. The naphthyl(di)imide unit was also identified by the absorption bands provided by the asymmetric stretch of the imidic C=O bond at 1712–1708 cm^−1^, the CN bond vibrations at 1321–1314 cm^−1^ and in-plane deformation of the imide ring at 780–756 cm^−1^. Whilst the absorption bands at 3080–3063 cm^−1^ and 1599–1592 cm^−1^ were associated with the vibrations of the aromatic C-H and C=C bonds, respectively, the aliphatic C-H bonds in the FTIR spectra of Cbz-based polymers, **P2** and **P4**, were evidenced by the absorption bands at 2959–2854 cm^−1^. Also, in the FTIR spectra of polymers **P3** and **P4**, the signature of the C-Cl bond was found through the absorption band at 935–932 cm^−1^. Figure 1 displays comparatively the recorded FTIR spectra of **P1**–**P4**.

### 3.2. Films Morphology

**Figure 2:**
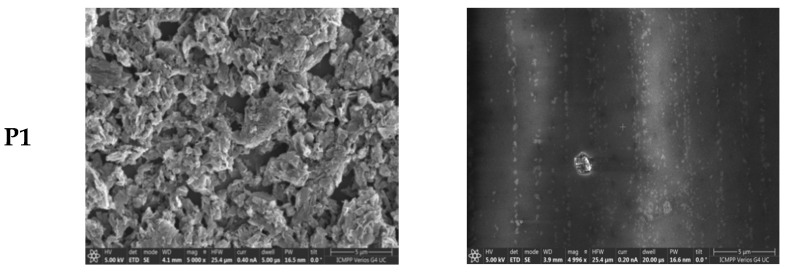

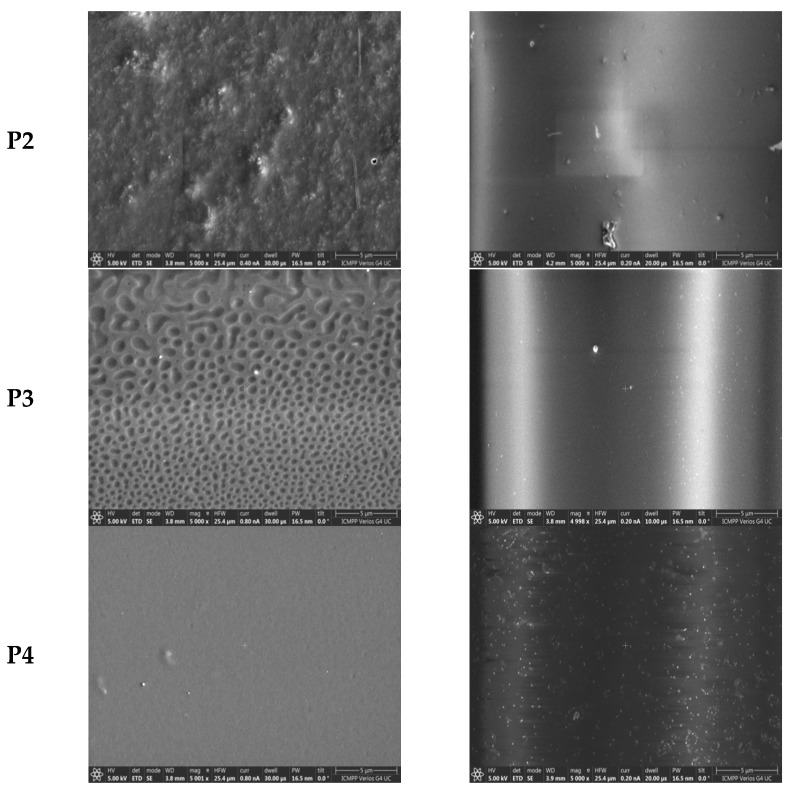
SEM images of **P1**–**P4** films deposited from chloroform solution by drop-casting (DC) and spin-coating (SC) techniques (scale 5 µm).

Generally, polymers can lead to defectless thin films, with flat and homogeneous surfaces, suitable for use as active layers in various applications, particularly in optoelectronics. This prerequisite is necessary to allow the current to flow through the device layers without producing short circuits. In this context, due to the good solubility in conventional organic solvents, **P1**–**P4** were processed into thin films on glass plates from CHCl_3_ solutions by using two wet-deposition techniques: spin-coating (SC) and drop-casting (DC). Scanning electron microscopy (SEM) was employed to examine the quality and morphology of the obtained films. According to SEM images shown in Figure 2, these polymer films are morphologically different, suggesting that each selected structural building block affected in a particular way the solid-state packing of the macromolecules. In addition, the solid-state packing arrangement varied in dependence on the deposition method.

Whereas the cast **P1** film consists of polymer chains organized into self-assembled sheet-like building blocks of various shapes and sizes, the solid-state packing of **P2** led to a dense network of wires that tend further to form spherical shapes. It seems that the bulk aliphatic side groups grafted on carbazole unit in **P2** induced a steric effect, reducing the packing ability of the polymer chains during the film preparation. When spin-coating was employed to obtain polymer films, these morphological entities were less distinct, while their evolution stopped at an intermediary phase, mainly due to the fast solvent evaporation. Thus, the solvent evaporation rate affected the polymer packing, since the macromolecules require time to rearrange and pack into specific assemblies. In the case of more flexible polymer chains (**P3** and **P4**), the macromolecular packing ability significantly decreased even in the case of drop-cast polymer films. During **P3** film preparation, the solvent evaporation led to isolated islands that protuberate out of the whole matrix. Even though the naphthyldiimide segment is a bulky unit, the TPA moiety allows for greater conformational changes of **P3** polymer chains than the Cbz unit by rotating around the azomethine bond and thus facilitating a mound-like morphology. Instead, the **P4** film showed a smooth morphology with no specific molecular arrangement, despite the better chain planarity induced by Cbz compared to TPA. From this perspective, it is assumed that the decreased ability to pack into tight structures originates from the presence of side bulky aliphatic chains in **P4** that cause a steric effect, preventing the tight molecular packing. Unexpectedly, the spin-coated film of **P4** displayed grain-like morphology, with small spherical structures surrounded by the amorphous polymer chains. In conclusion, it should be stated that TPA moiety allows for significant macromolecular chain packing compared to the related Cbz-derived polymers, despite the more rigid structure of this heterocycle, mostly due to the great effect exerted by the lateral aliphatic groups.

### 3.3. Thermal Stability

**Figure 3:**
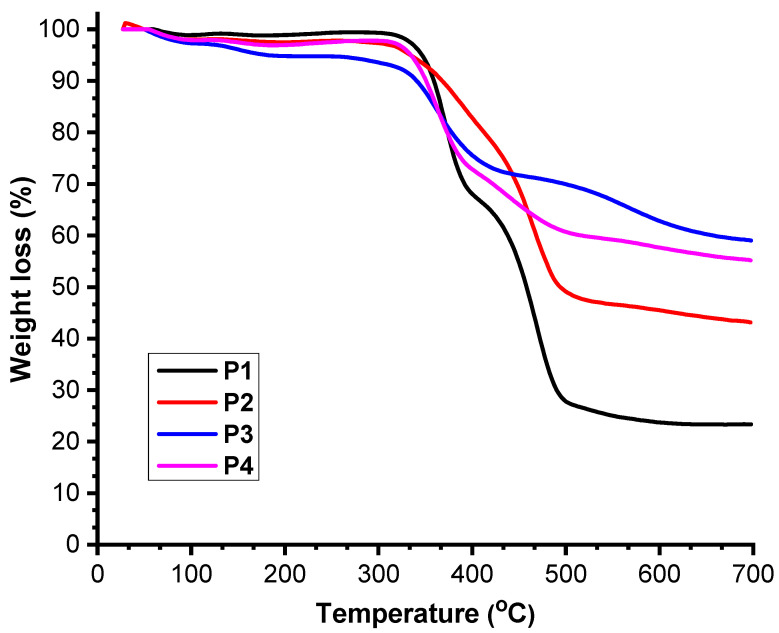
TGA curves of naphthyl(di)imide-based polymers **P1**–**P4**.

Thermogravimetric analysis (TGA) was involved as an experimental tool to evaluate the thermal stability of the present polymers up to 700 °C. The recorded TGA curves for **P1**–**P4** are displayed in Figure 3.

All investigated polymers present high thermal stability, with decomposition temperatures well above 318 °C. According to the TGA curves, **P1** is the most thermally stable in the series (345 °C), being followed by **P4** (338 °C), **P3** (328 °C) and **P2** (318 °C). Although **P1** and **P3** contain TPA as a structural element, which usually endows polyimides with high thermostability [20,29], **P3** is less thermally stable than **P1.** This can be reasonably explained by the presence of the more rigid and planar naphthyldiimide in the structural unit of **P1**. This moiety appears to exert a better effect on thermal stability than on the two naphthylimide moieties in **P3**, despite the higher molecular weight of the latter. In addition, the twisting CCl_2_ center in the dianhydride-derived fragment of **P3** lowers the polymer planarity, thus overcoming the impact induced by the molar mass on thermal stability. The Cbz-based polymer **P2** decomposes at a significantly lower temperature than the related polymer **P1** containing TPA, which is an expected behavior in light of the presence of aliphatic side chains in **P2**, which are less thermally stable, thus reducing the overall polymer thermostability. Compared to **P3,** a slightly higher thermal stability was noticed for **P4**, although the opposite effect was expected due to the presence of the aliphatic groups-substituted Cbz. It appeared that the higher molecular weight of **P4** compensates the thermal effect induced by the aliphatic side chains, so that its thermal stability exceeds the one of the related TPA-based polymer **P3**. Any attempt to evaluate the glass transition temperature (T_g_) of these polymers failed. No detctable T_g_ was registered up to 300 °C, suggesting their rigid nature, or even the presence of molecular interactions between macromolecules in a solid state.

### 3.4. Photophysical Studies

#### 3.4.1. UV-Vis Absorption Characteristics

**Figure 4:**
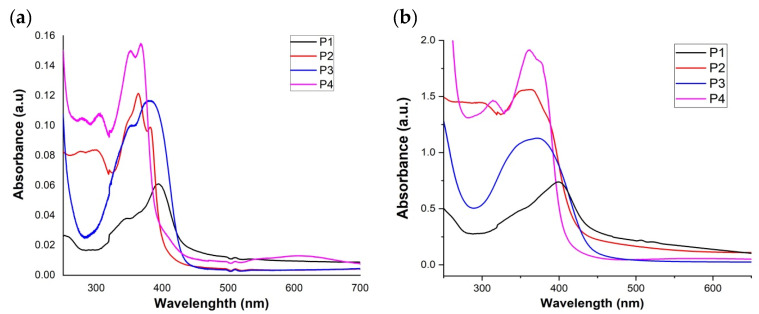
UV–Vis absorption spectra of **P1**–**P4** in (**a**) CHCl_3_ solution and (**b**) thin films cast from CHCl_3_ solution.

The ultraviolet-visible (UV-Vis) absorption response of polymers **P1**–**P4** was first investigated in CHCl_3_ solutions (10^−5^ M) and then in thin films drop-cast from CHCl_3_ solution. The recorded spectra are comparatively shown in Figure 4, whereas the optical data extracted from these spectra are listed in Table S1.

As a whole, it was noticed that the spectral pattern in a solution of TPA-containing polyazomethines **P1** and **P3** consists in only one band system, whereas the Cbz-based polymers **P2** and **P4** display a spectra with two band systems (Figure 4a). The supplementary absorption bands with maxima at 275/278 and 300/306 nm recorded in the case of **P2** and **P4** were attributed to the π-π* transitions in the Cbz chromophore [30]. The lower energy absorptions centered at 347/352, 364/368 and 382/407 (**P2**/**P4**) can be associated with both the vibronic states of the naphthyl(di)imide segment and π-π* transitions of azomethine moiety or carbazole–azomethine conjugated segments. Since the azomethine and naphthyl(di)imide are electron-withdrawing units and Cbz an electron-donor moiety, a slight internal charge transfer is expected to occur that can also contribute to the absorption band system registered at higher wavelengths. A distinct feature in the spectrum of **P4** is, however, the appearance of a very low energy band, with a maximum at 604 nm, which can be accurately associated with the direct HOMO to LUMO transition, as predicted by DFT simulations on the corresponding oligomer (**Az3**), reported in reference [28]. As seen in Figure 4, the UV–visible absorption bands from the highest wavelengths of **P1** and **P3** containing TPA are red-shifted (approx. 15 nm) and broadened compared with those of related Cbz-based polymers, whereas their origins can be found in both π-π* transitions occurring in naphthyl(di)imide, azomethine and TPA–azomethine fragments, as well as in ICT transitions between donor and acceptor units. Such behavior suggests a better electron transfer in the case of TPA-based polymers due to the stronger ability of TPA to push electrons to the acceptor units compared to Cbz.

The solvent change from CHCl_3_ to the one with higher polarity (DMF) revealed some small hypsochromic shifts only for the lowest energy absorption band, the maximum shift of 6 nm being registered in the case of **P1** (Figure S3). This slight negative solvatochromic behavior that suggests a better stabilization by solvation of molecules in ground state relative to excited state with decreasing solvent polarity provides clear evidence for the ICT character of the transitions contributing to this absorption.

In thin films, all polymers preserved the UV–Vis absorption spectral profiles of the macromolecules in solution (Figure 4b). The only difference relates to the position of the absorption maxima that are red-shifted with respect to the corresponding absorptions of the isolated molecules in solution. These bathochromic shifts are mostly induced by the intermolecular packing of polymer chains in solid state, when macromolecules can adopt more planar conformations than in solution, leading to an extended conjugation. In addition, the HOMO to LUMO transitions in the spectrum of **P3** are no longer present, being suppressed by the molecular rearrangement in solid state. 

#### 3.4.2. Fluorescence Characteristics

**Figure 5:**
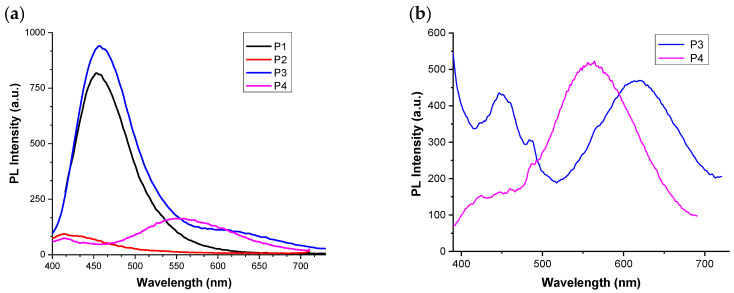
Fluorescence spectra of **P1**–**P4** in (**a**) CHCl_3_ solution and (**b**) thin films cast from CHCl_3_ solution.

The fluorescence spectra of the synthesized polymers recorded in CHCl_3_ solutions by excitation with wavelengths corresponding to the absorption maxima indicated a strong influence of the structural parameters on the emissive properties. According to the fluorescence spectra shown in Figure 5, the TPA-based polymers **P1** and **P3** displayed fluorescence spectra with a well-defined intense emission band centered at 453 nm and 456 nm, respectively, which can originate from the naphthyl(di)imide that is usually a blue-light emissive fluorophore [26,31]. Apart from this, a weak emission occurred in the spectrum of **P3** at 626 nm, which can be associated with the ICT emission (Table S1). It is worth nothing that the presence of TPA in the structural pattern of **P1** and **P3** prevented the quenching of monomer emission usually encountered at polyazomethines [6,32]. This is the case with carbazole-based polyazomethines **P2** and **P4,** which are weakly emissive due to the lack of monomer emission, either provided by carbazole or naphthyl(di)imide.

Although it was expected that Cbz would substantially improve the intrinsic emission of these polymers, in fact it contributed to the almost complete fluorescence suppression. Previous investigations by fluorescence spectroscopy and laser flash photolysis revealed the absence of triplet states, providing the conclusion that the azometine bond induces the quenching of the singlet excited state [6]. The fluorescence deactivation modes were associated with both the rotation around the aryl-N linkage and intramolecular photoinduced electron transfer to the azomethine [33,34]. However, in the case of **P4**, a weak emission band with a maximum at 555 nm was recorded, being attributed to the ICT state’s emission.

The influence of solvent polarity on fluorescence properties of **P1**–**P4** has been also explored by recording the spectra in three additional solvents: DMF, tetrahydrofurane (THF) and N-methylpyrrolidone (NMP). By polarity increase, the ICT emission is no longer allowed, whereas the intrinsic fluorescence band profiles are less resolved (Figure S4). Whereas the PL spectra of **P1** and **P3** in THF mirror the ones obtained in CHCl_3_ with small shifts, in DMF and NMP these polymers displayed red-shifted emission bands, with two maxima (**P1**, DMF: 445, 476 nm; NMP: 460, 483 nm) or one shoulder-like maximum and one maximum (**P3**, DMF: 428, 480 nm; NMP: 483, 530 nm). These can be tentatively ascribed to the H-bond formation between these polymers and highly polar solvent molecules [17], leading to the loss of polymer coplanarity compared to that in CHCl_3_, and the appearance of twisted locally excited states. Thus, in the case of TPA-based polymers, the overall polymer emission can arise from mixed states: planar and twisted excited states. Note that Cbz-based polyazomethine **P4** also exhibited fluorescence in DMF, the emission maximum being centered at 464 nm, whereas in the case of **P2** the fluorescence was completely quenched. We may assume that the intramolecular photoinduced electron transfer is no longer possible in the twisted conformer of **P4** formed in DMF, and, as a consequence, the locally excited states energy is transferred to the ground state. On the other hand, the molecular twisting of the more rigid Cbz-based related polymer **P2** is not strong enough to prevent the internal conversion due to the rotation around the azomethine bond or the photoinduced electron transfer to azomethine.

However, a particular florescence response was noticed in thin films obtained from CHCl_3_ solution (Figure 5b). Indeed, the most intense emission was obtained at high wavelengths, and only in the case of naphthylimide-based polymers **P3** and **P4**. The emission band perfectly overlaps with the ICT emission band registered in CHCl_3_ solution for each polymer; therefore, it can be excluded that it arises from aggregates or excimers. However, the spectrum of the TPA-based polyazomethine **P3** also contains the contribution from the locally excited states’ emission, which is expected in the view of the most twisted conformation of this polymer in the investigated polymer series.

#### 3.4.3. Optical Bandgap Energy

The bandgap energy (E_g_) and the position of the highest occupied molecular orbital (HOMO) and the lowest unoccupied orbital molecular (LUMO) *versus* vacuum are among the most important parameters of the donor-acceptor polymers. By structural design, the bandgap and the position of HOMO and LUMO levels can be finely tuned, as was our target, too. An accurate estimation of the bandgap of an organic semiconductor is often challenging. The optical gap is usually associated with the energy of the onset of electronic absorption band that can be accurately evaluated by the Tauc method [35], according to the relation:

αhν ∝ (hν − E_g_)^n^ (1)

where E_g_ is the optical band gap energy, h is the Planck’s constant, ν is the frequency of light, n is a factor that characterizes the electronic transition mode (direct/indirect and allowed/forbidden) and α is the absorption coefficient.

The absorption coefficient against the photon energy—(αhv) *versus* hv dependence allows estimating the edge of the electronic absorption spectrum from the intersection of the linear fit with the hv axis. According to previous studies [36,37], n was considered equal to ½. The obtained E_g_ values for the investigated polymers are listed in Table 2. These were found to range from 1.8 to 2.9 eV, being largely dependent on the molecular structure but also on the solid-state packing arrangement of the individual molecules in thin films.

By far, the polyazomethine with the lowest bandgap energy is **P1**, containing naphthyldiimide and TPA moieties that induce a simple conformational chain packing, which favors electron delocalization and conjugation along the backbone. In addition, the strong intermolecular interactions taking place via stacking of individual **P1** molecules, as observed in SEM images, mostly contributed to a better electron delocalization between polymer chains or exciton coupling [38,39], leading to the decrease of the bandgap energy. The incorporation of alkyl-substituted Cbz units in the **P2** backbone was less efficient in lowering the bandgap, although Cbz is more planar than TPA. This is mainly due to the interchain effect’s suppression, which hindered the molecular order. The bulky side chains enhance the spatial distance between the macromolecules and prevent their interaction. Such disorder alters the charge carrier’s mobility in polymer films, with a direct impact on the bandgap energy, but contributes to a better solubility and processability of the polymers by wet methods. Most likely, by grafting of shorter alkyl groups on Cbz moiety, a compromise between solubility and solid-state organization can be achieved. A similar effect on the bandgap energy was noticed when multiple twisting centers are present along the chains, as in the case of **P3** and **P4**. These reduced the overall conjugation and path for electrons’ delocalization in these polymers. As expected, the effect is greater in **P4** due to the presence of alkyl chain spacers, both effects contributing to the E_g_ increase.

### 3.5. Polymer’s Electroactivity and Electronic Structure

**Figure 6:**
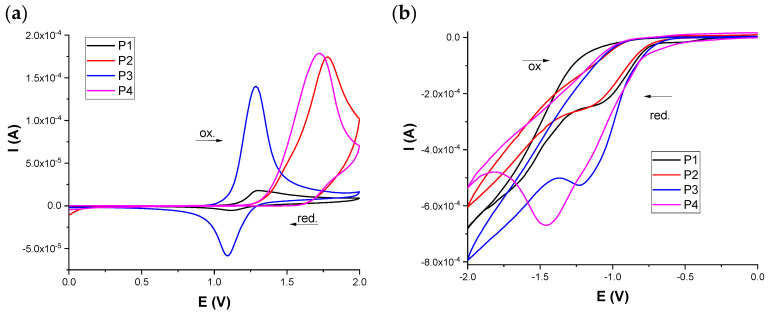
The CV curves of **P1**−**P4** films coated on ITO electrodes in (**a**) anodic and (**b**) cathodic regions.

The electroactivity of **P1**–**P4** in solid state was investigated by cyclic voltammetry (CV) upon current sweep between 0 and 2 V to survey their capacity to release electrons, as well as in the potential window between 0 and −2 V to evaluate their electron *pulling* ability. For this purpose, polymer-modified ITO glass plates were used as working electrodes in electrochemical cells fitted with Pt wire and Ag/Ag^+^ as counter and reference electrodes, respectively, and tetrabuthylammonium perchlorate in ACN (0.1 M) as supporting electrolyte. Figure 6 exhibits the CV waves recorded for **P1**–**P4** in the anodic and cathodic regions, at a scan rate of 50 mV/s. The electrochemical data extracted from the CV curves are listed in Table 3.

According to Figure 6a, all polymers are electroactive in the positive region and can be oxidized in quasi-reversible (**P1** and **P3**) or irreversible (**P2** and **P4**) processes. The redox couple recorded for **P1** and **P3** at the potential values presented in Table 3 can be accurately assigned to the quasi-reversible oxidation of the central N atom of the TPA unit to radical cations. The oxidation potential is slightly influenced by the ability of the imide core to withdraw electrons. Thus, **P1** is very difficult to oxidize compared to **P3** due to the stronger *pulling* effect exerted by naphthyldiimide in **P1** compared to the one of two naphthylimide fragments in **P3**. A similar trend was observed in the case of Cbz-based polyazomethines **P2** and **P4**. However, the Cbz heterocycle underwent a very difficult oxidation process compared to TPA, denoting the difficulty of *pushing* electrons in these polymer systems. In addition, the formed radical cations generated under irreversible conditions are unstable, their restoration to the neutral forms being completely prevented by the electron-withdrawing azomethine–naphthylimide framework.

No redox couple was recorded in the negative potential window, although a slight tendency was noticed. Here, only one reduction peak was clearly seen and attributed to the reduction of the naphthyl(di)imide cores of the polymers, according to previous reports [40]. Although the reduction of azomethine was expected to occur at potentials below −2V, the second reduction observed in the CV curve for **P1** can be associated with the formation of azomethine radical anions, or the reduction of bisimide anion to the dianion form. After the first reduction, the electron density on the O atom of imide carbonyl became greater, reducing the ability of the radical anion to accept an additional electron [41]. Still, when the extra electron density can be delocalized on the aromatic cores, as in the case of naphthyldiimide, the second reduction is made possible by inserting the additional electron at the other imide carbonyl or azomethine. The absence of this process in the case of **P2** can be explained by the lower capability of Cbz to *push* electrons compared to TPA. The first reduction takes place at similar potential in **P1** and **P2**, being barely affected by the electron donor unit. Instead, the naphthylimide core in **P2** and **P4** is very difficult to reduce to the radical anion state, and at significantly different potentials. It is obvious that TPA, with its stronger ability to release electrons than Cbz, induces an easier reduction of the naphthylimide core. The second reduction step was not detected in the studied potential window, probably due to the impossibility of the smaller size naphthylimide moiety to stabilize the dianion form.

**Figure 7:**
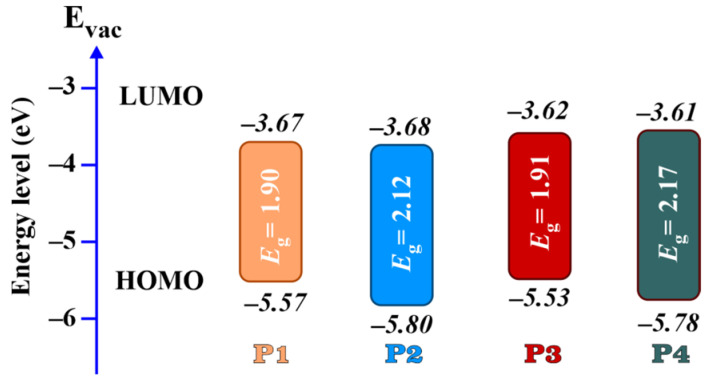
HOMO, LUMO and bandgap energy diagram of **P1**–**P4**.

The suitability of the synthesized polymers for use as active elements in optoelectronic devices can be discussed on the basis of HOMO, LUMIO and bandgap energies. To evaluate these characteristics, the first oxidation and reduction onset potentials were used by assuming that the position of the Fc/Fc^+^ redox system used for calibration is 4.78 eV *versus* vacuum level. Thus, according to the well-known relations [27,42] and the onset potential of Fc/Fc^+^ measured in our system against Ag/Ag^+^, which is 0.34 eV, an estimation of the above-mentioned parameters was made, as shown in Table 3. From this table, it is easy to observe that the LUMO energy is considerably affected by the *pulling* ability of the imide–azomethine framework and is slightly influenced by the electron-donor unit capability. Instead, the HOMO energy is significantly dependent on the electron-donor strength, the exchange of TPA with Cbz leading to an energy variation of approx. 0.23–0.25 eV. The synergistic effect of the electron donor and acceptor units in polymers **P1**–**P4** led to a bandgap energy variation from 1.9 eV to 2.17 eV (Figure 7), placing them among electroactive materials with both electron- and hole-transporting characteristics that are suitable for opto-electronic applications. However, there is a difference between the electronic and optical bandgap values, as expected, due to their different means (minimal energy required to create an electron hole pair in a semiconductor *versus* the exciton energy that determines the onset of vertical interband transitions) and distinct experimental techniques [43].

## 4. Conclusions

Novel polynaphthylimide-azomethines with donor-acceptor architecture were synthesized and thoroughly investigated to anticipate their potential use as electroactive materials in energy or optoelectronic applications. The constituent units, which are naphthyl(di)imide, azomethine and Cbz/TPA, beside the direct connection of imide with azometine through the N-N bond, allowed the fine tuning of the solid-state packing and overall physicochemical characteristics of the polymers. The naphthyl(di)imide moiety endowed the azomethines with high thermal stability, up to 318 °C, while it dictated the overall rigid shape of the molecules. Still, all the investigated oligomers displayed a good solubility, which allowed their processing in thin films of various morphologies. In the UV-Vis absorption spectra, the signatures of both localized and ICT transitions were identified, denoting a complex optical behavior. Thus, the present polymers displayed light emission from localized or ICT transitions depending on the experimental conditions, donor-acceptor strength and polymer chain geometry. The cyclic voltammetry analysis evidenced variable hole- and electron-transport capability, with a direct impact on the HOMO, LUMO and bandgap energies. With electrical and optical characteristics well-modulated by the molecular design, the investigated polymers can be considered the starting point for designing new donor-acceptor systems by interplay between donor and acceptor units, which may find applications as active layers in energy-related devices or other optoelectronic applications.

## Figures and Tables

**Table 1:** Molecular weights (evaluated by GPC in CHCl_3_) and TGA data of **P1**–**P4**.

Polimers	M_n_ (g/mol)	M_w_ (g/mol)	M_w_/M_n_	T_onset_ (°C)
**P1**	1280	1540	1.19	345
**P2**	2150	2880	1.34	318
**P3**	2700	5100	1.88	328
**P4**	3290	5660	1.72	338

**Table 2:** Optical band gap data of for polymer films estimated by the Tauc method.

Polymer	E_g_ (eV)	Thickness (µm)
**P1**	1.8	0.512
**P2**	2.7	0.351
**P3**	2.6	0.638
**P4**	2.9	0.357

**Table 3:** The electrochemical characteristics of polyazomethines **P1**−**P4** films.

Polymer Film	Oxidation Potential (V)		Reduction Potential (V)		Energy (eV)		
	E^ox^_onset_	E^ox^	E^red^_onset_	E^red1^	E_LUMO_	E_HOMO_	E_g_
**P1**	1.13	1.30	−0.77	1.12 −1.07 −1.61	−3.67	−5.57	1.9
**P2**	1.36	1.78	−0.76	−1.13	−3.68	−5.8	2.12
**P3**	1.09	1.29	−0.82	1.09 −1.23	−3.62	−5.53	1.91
**P4**	1.34	1.73	−0.83	−1.46	−3.61	−5.78	2.17

E_onset_—onset potential; E^ox^—anodic peak potential; E^red^—cathodic peak potential; E_g_—bandgap energy.

## Data Availability

The data presented in this study are available on request from the corresponding author.

## Supplementary Materials

The following supporting information can be downloaded at: https://www.mdpi.com/article/10.3390/molecules27185761/s1, Figure S1: Structures of the monomers (diamines and dialdehyde); Figure S2: 1H–NMR spectra of **P1**–**P3** in DMSO-d6 and **P4** in CDCl_3_; Figure S3: Comparative UV-vis absorption spectra of **P1** in CHCl_3_ and DMF solutions; Figure S4: Fluorescence spectra of **P1**—**P4** in solvents of different polarities; Table S1: Optical data of polyazomethines **P1**—**P4**.
