# Supplementary material for: Polynaphthylimide–Azomethines Containing Triphenylamine or Carbazole Moieties with Tuned Optoelectronic Properties through Molecular Design
Source: Molecules. 2022 Sep 6;27(18):5761. doi: 10.3390/molecules27185761 (PMC9504240; doi:10.3390/molecules27185761)
Supplement: Supplementary file 1 [file molecules-27-05761-s001.zip › molecules-1850752-supplementary.pdf]

Supporting Information (SI)

for

Polynaphthylimide-Azomethines containing Triphenylamine or Carbazole Moieties with Tuned Optoelectronic Properties through Molecular Design

Marius SOROCEANU, Catalin-Paul CONSTANTIN, and Mariana-Dana DAMACEANU*

"Petru Poni" Institute of Macromolecular Chemistry, Aleea Gr. Ghica Voda 41A, Iasi-700487, Romania

* Corresponding author, email: damaceanu@icmpp.ro

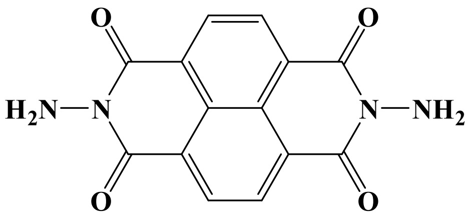

M1

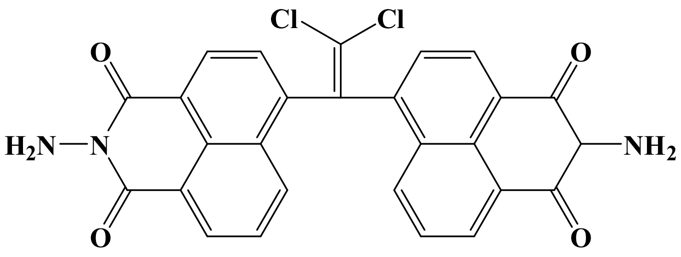

M2

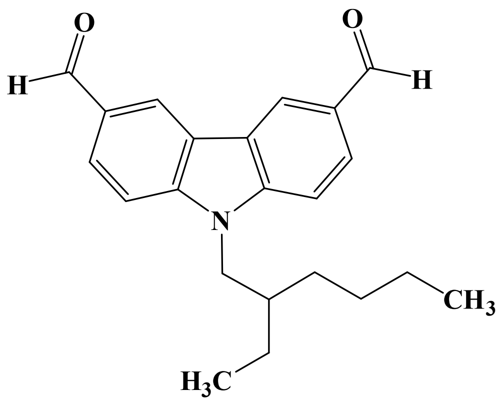

M3

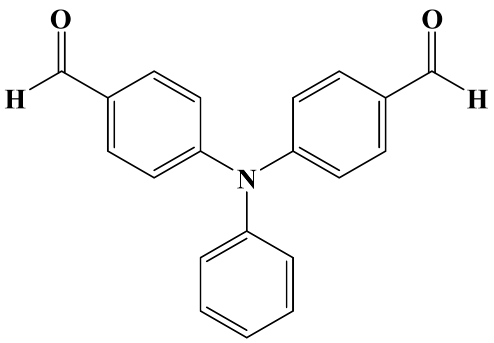

M4

Figure S1. Structures of the monomers (diamines and dialdehyde)

a)

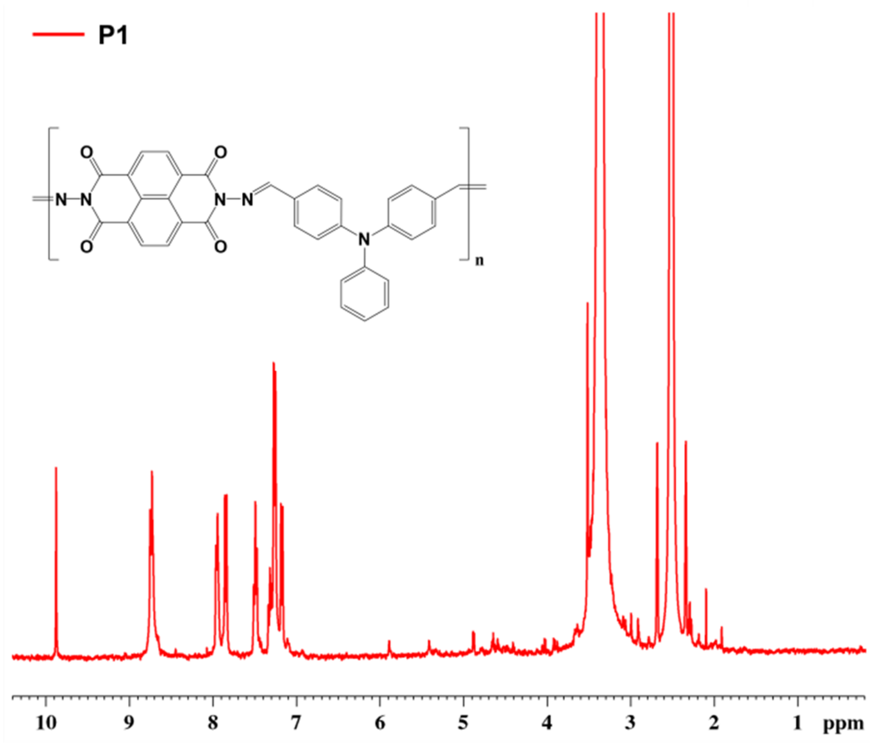

b)

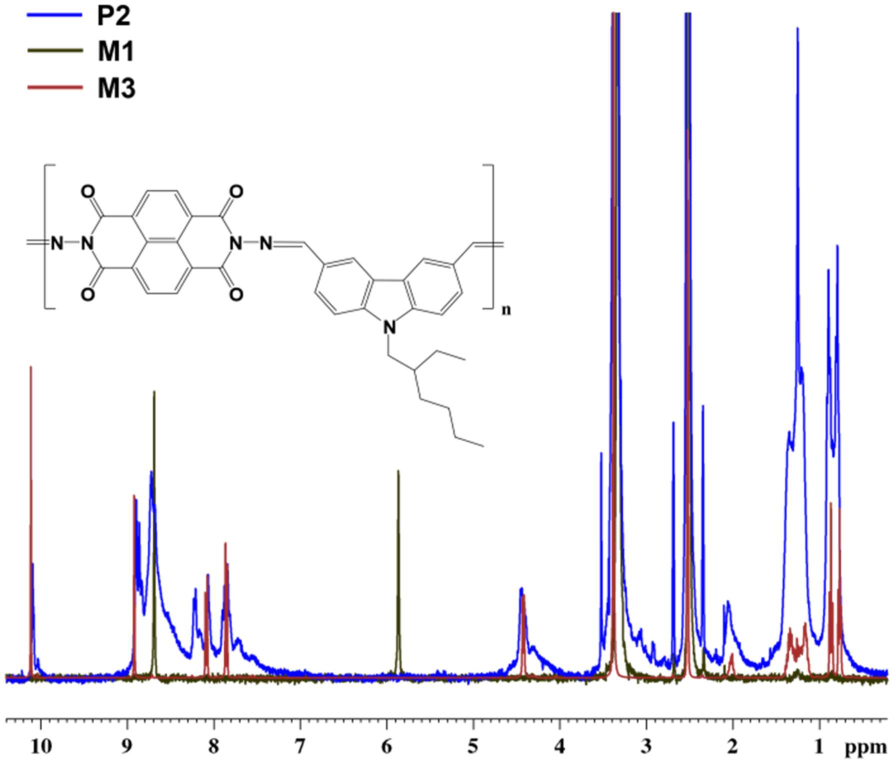
P2
M1
M3
O
O
O
O
N
N
N
N
N
n
10
9
8
7
6
5
4
3
2
1
ppm

c)

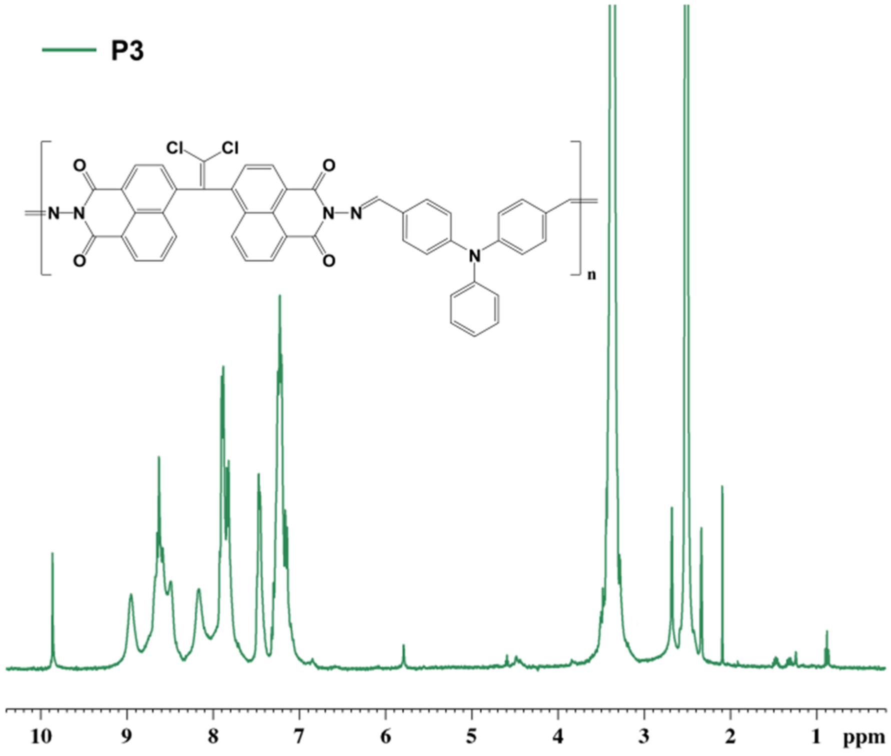
P3
Cl
Cl
O
O
O
O
N
N
N
N
N
n
10
9
8
7
6
5
4
3
2
1
ppm

d)

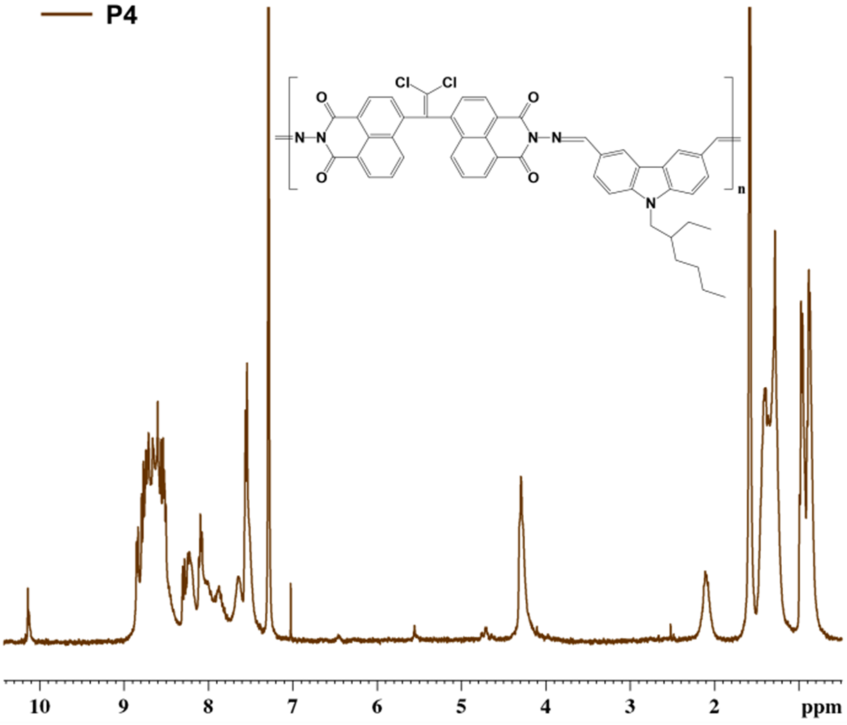

Figure S2. ¹H–NMR spectra of **P1–P3** in DMSO-d_6 and **P4** in $CDCl_3$

Table S1. Optical data of polyazomethines **P1-P4**

Polymer	Solution				Film	
	λ_{abs} (nm)		λ_{em} (nm)		λ_{abs} (nm)	λ_{em} (nm)
	$CHCl_3$	DMF	$CHCl_3$	DMF		
**P1**	321^{s}, 344^{s}, 363^{s}, 394	291, 321^{s}, 346^{s}, 366^{s}, 389	453	445, 476	320^{s}, 348^{s}, 373^{s}, 399	-
**P2**	275, 300, 347^{s}, 364, 382	277, 297, 348, 363, 381	415^{w}	409^{w}	273, 301, 351, 366, 387^{s}	-
**P3**	314^{s}, 334^{s}, 352, 381	292s, 334s, 348s, 375	456, 626^{w}	428^{s}, 480	335s, 354s, 379, 400^{s}	448, 486^{s}, 617
**P4**	278, 306, 352, 368, 407^{s}, 604^{w}	277, 299, 350, 365	$415^{w,}$ 555	411^{w}, 464	315, 361, 378	561

λ_{abs} - maximum absorption wavelength; **λ_{em}** - maximum PL emission wavelength; s – wavelength corresponding to the shoulder-like peak; w – weak.

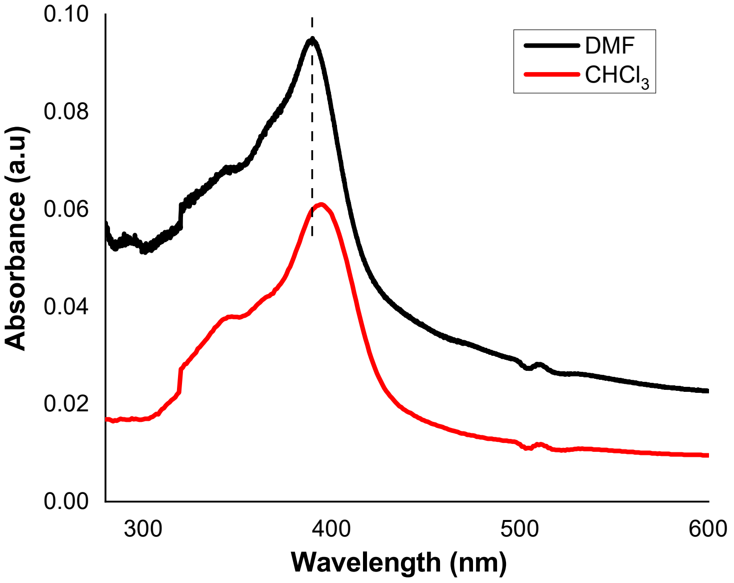

Figure S3. Comparative UV-vis absorption spectra of **P1** in $CHCl_3$ and DMF solutions

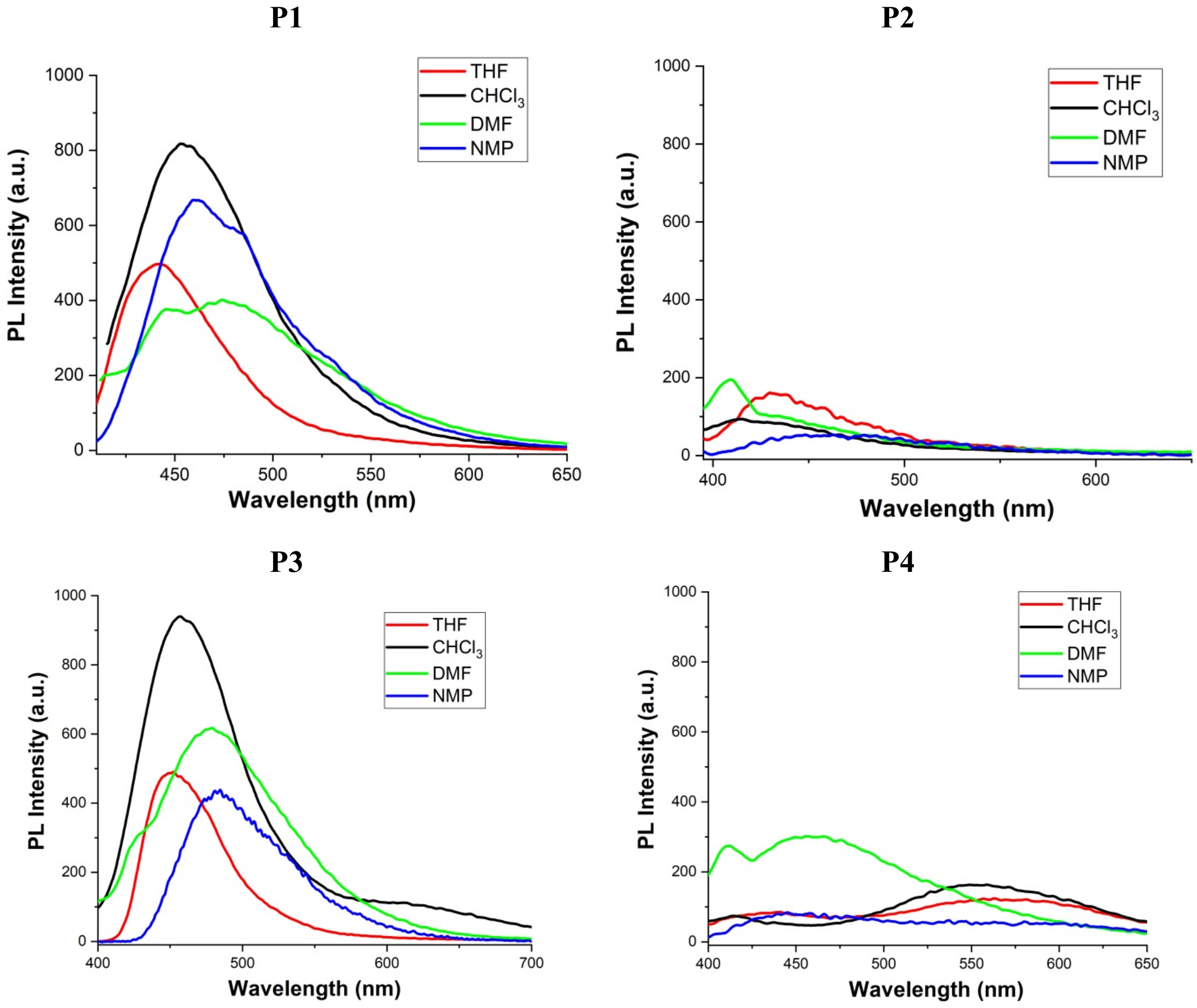

Figure S4. Fluorescence spectra of **P1—P4** in solvents of different polarities
